# Ciliary Body Seeding after Pars Plana Transvitreal Fine-Needle Aspiration Biopsy of Choroidal Melanoma

**DOI:** 10.18502/jovr.v15i2.6744

**Published:** 2020-04-06

**Authors:** Jideofor K. Ndulue, Arman Mashayekhi, Carol L. Shields

**Affiliations:** ^1^ Wills Eye Hospital, Ocular Oncology Service, Philadelphia, PA, USA

**Keywords:** Choroid, Ciliary Body, Extraocular Extension, Fine-Needle Aspiration Biopsy, Melanoma, Seeding

## Abstract

**Purpose:**

To report ciliary body seeding 20 years after pars plana transvitreal fine needle aspiration biopsy (FNAB) of choroidal melanoma.

**Case report:**

67-year-old man with choroidal melanoma in left eye was previously managed with pars plana FNAB using a 25-gauge needle followed by plaque radiotherapy. Twenty years later, choroidal melanoma was regressed but there was a small flat focus of scleral pigment 3.0mm from the limbus at the FNAB site. Ultrasound biomicroscopy showed a contiguous ciliary body mass measuring 3.1mm in thickness. Tumor seeding in the anterior chamber angle was noted inferiorly. These findings suggested melanoma recurrence along the needle tract. Treatment was performed with Iodine-125 radioactive plaque covering entire anterior segment and ciliary body recurrence. The tumor regressed to 2.2mm over one year.

**Conclusion:**

Pars plana transvitreal FNAB of choroidal melanoma resulted in needle tract seeding in ciliary body and episcleral region 20 years later.

##  INTRODUCTION

The diagnosis of uveal melanoma is usually based on ophthalmoscopic appearance and results of noninvasive tests, such as ultrasonography, transillumination, fluorescein angiography, and optical coherence tomography.^[1,2]^ Fine-needle aspiration biopsy (FNAB) is occasionally performed to obtain a cytopathologic confirmatory diagnosis of suspected but atypical uveal melanoma.^[1,2][3]^ Moreover, over the past two decades, FNAB has been used extensively for prognostication of uveal melanoma based on the results of cytogenetic testing and gene expression profiling. Genetic testing has markedly increased the number of FNABs being performed in eyes with uveal melanoma.^[1,2][3]^

FNAB for choroidal melanoma, depending on the tumor location, can be performed safely with the direct transscleral approach into the tumor base or pars plana transvitreal approach into the tumor apex.^[1,2][3]^ Transient localized subretinal/preretinal or intravitreal hemorrhage at the tumor biopsy site is the most common complication of this procedure.^[1,2][3]^ Seeding along the needle tract is a rare complication.^[4,5,6,7]^

Here, we report a case of melanoma seeding along the needle tract in the ciliary body and extraocular tissue at the pars plana needle entrance site 20 years after pars plana transvitreal FNAB of choroidal melanoma.

##  CASE REPORT

A 67-year-old Caucasian man was referred to the Oncology Service, Wills Eye Hospital, for an evaluation of a peripheral iris elevation at the 9:00 o'clock position in the left eye (OS). He had a history of choroidal melanoma OS, confirmed using FNAB and treated with iodine-125 plaque brachytherapy 20 years before referral. In the FNAB, a 25-guage needle bent to an 85° angle was inserted via a 1.5-mm partial-thickness scleral incision approximately 3.5 mm posterior to the limbus OS along the 10:00 o'clock meridian. Under indirect ophthalmoscopy, the needle was advanced through the vitreous cavity into the choroidal mass, and aspiration was performed. After aspiration, mild vitreous hemorrhage was noted, and hemostasis was achieved by applying direct pressure on the globe. A nylon suture was used to close the scleral incision site. Cytological examination of the aspirate showed mixed spindle and epithelioid melanoma cells.

On examination at our center, 20 years later, visual acuity was 20/20 in the right eye (OD) and 20/25 OS. Intraocular pressure was 9 mmHg OD and 11 mmHg OS. The right eye was unremarkable.

**Figure 1:**
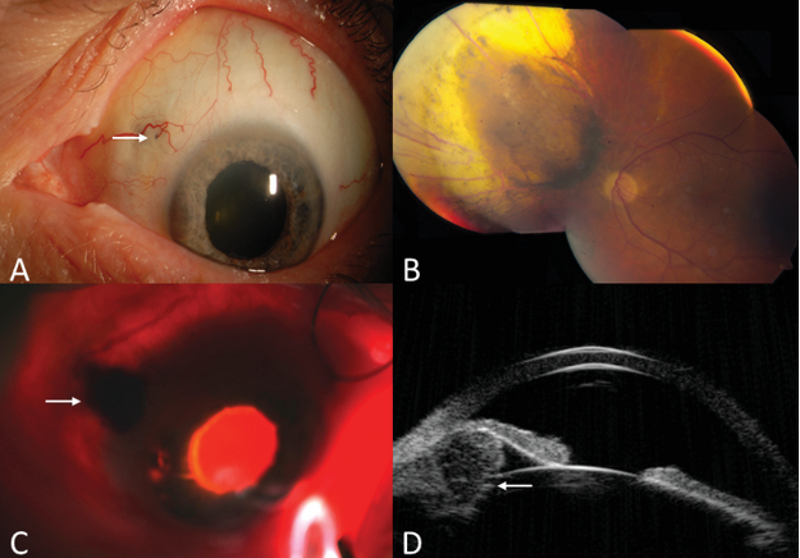
(A) Dilated episcleral blood vessel superonasally perforates the sclera 3mm from the limbus with a pigmented spot of extrascleral extension (arrow). Additional invasion of the peripheral iris is noted nasally. (B) Fundus photograph of the same eye shows irradiated choroidal melanoma with surrounding retinal pigment epithelium atrophy. (C) Transillumination demonstrates a dark shadow in the pars plana region (arrow). (D) Ultrasound biomicroscopy shows a ciliary body mass measuring 3.1mm in thickness with bowing forward of the peripheral iris.

The OS had a superonasal dilated episcleral blood vessel that perforated the sclera 3 mm from the limbus [Figure 1(A)]. A flat, darkly pigmented episcleral mass, measuring 0.2 mm in the basal diameter was noted at the 10:00 o'clock meridian at the site of entry of the episcleral vessel [Figure 1(A)]. The iris was elevated nasally along the 9:00 o'clock meridian. Gonioscopy revealed confluent seeding in the inferior angle and multiple tiny seeds over the temporal and superior trabecular meshwork. Funduscopy of the left eye revealed a flat retina with regressed choroidal melanoma measuring 8.0 × 6.0mm in basal dimensions and 1.9mm in thickness. The tumor was surrounded by an area of retinal pigment epithelium atrophy, and nasal retinal vessels had occlusion [Figure 1(B)].

Transillumination of the ciliary body revealed a superonasal dark shadow in the pars plana [Figure 1(C)]. Ultrasound biomicroscopy demonstrated a ciliary body mass of 3.1mm thickness at the site of the shadow, causing forward bulging of the peripheral iris [Figure 1(D)]. Based on these findings, a clinical diagnosis of ciliary body melanoma with anterior chamber invasion, angle seeding, and early extraocular extension was made.

The patient was treated with a round 18mm custom-designed ${}^{125}$ I radioactive plaque covering the entire anterior segment and delivering a dose of 7,000 cGy to the tumor apex at a depth of 4.0 mm. FNAB of the ciliary body tumor for cytogenetic evaluation at the time of plaque placement showed a loss of 1p, disomy 3, gain of 6p, and disomy 8. At the one-year follow-up, choroidal melanoma had remained regressed at 1.9mm thickness, and ciliary body melanoma had regressed to 2.2mm thickness. The small extrascleral component remained stable with no evidence of orbital recurrence on clinical examination or orbital imaging. Systemic evaluation showed no evidence of systemic metastasis.

##  DISCUSSION

FNAB was initially used to obtain tissue for cytopathologic diagnosis of atypical tumors in the management of choroidal melanoma. Recently, its use has expanded to cytogenetic prognostication of the metastatic risk.^[1,2][3]^ Therefore, the use of FNAB for choroidal melanoma has increased at many centers. A recent study on reactions to and desire for prognostic testing for choroidal melanoma showed that even in the absence of prophylactic therapies to improve prognosis, 97% of respondents preferred knowing prognostic information.^[8]^

A serious potential complication of FNAB is extraocular tumor extension. Nonetheless, several studies proved the safety of FNAB for choroidal melanoma with the transscleral or pars plana transvitreal approach,^[1,2][3]^ and cases of extraocular extension are rare.^[4,5,6,7]^ Schefler et al^[4]^ reported extraocular extension in four patients with uveal melanoma, but three of them had undergone vitrectomy and/or open biopsy in addition to FNAB. The interval between FNAB and the detection of extraocular extension in their series ranged from five months to approximately nine years. Caminal et al^[5]^ reported epibulbar tumor seeding eight months after pars plana transvitreal FNAB in one patient using a 25-gauge needle and infusion. Mashayekhi et al^[6]^ reported one patient with extraocular extension of ciliochoroidal melanoma one and a half years after transscleral FNAB using a 25-gauge-needle without infusion. Koch et al^[7]^ reported pars plana seeding with localized extraocular extension 3.5 years after transretinal biopsy of choroidal melanoma using a 25-gauge vitrectomy probe followed by Ruthenium-106 plaque radiotherapy.

Laboratory quantification of tumor seeding following FNAB of the enucleated globes with uveal melanoma showed significantly fewer tumor cells in the needle tract of the pars plana transvitreal or translimbal approach compared to the transscleral approach.^[9]^ Other factors possibly associated with a higher risk of tumor seeding via the needle tract include a larger needle gauge, higher number of needles passing into the tumor, vitreous leakage through the scleral entrance site, manipulation of the needle within the tumor, and a higher number of viable malignant cells aspirated. Additionally, fluid infusion into the vitreous cavity could theoretically increase the risk for tumor cell egress. Immediate application of cryotherapy at the scleral entrance site as the needle is removed and application of radioactive plaque over the scleral entrance site could prevent tumor seeding.^[4]^

The long interval of 20 years between FNAB and the detection of ciliary body seeding in our case is an indication of the low-grade nature of the treated choroidal melanoma. In addition, the location of seeding in the ciliary body, a region hidden from direct view, contributed to the delay in detection of seeding. Bosello et al reported delayed recurrence of uveal melanoma 45 years after treatment, possibly caused by the low malignant potential, tumor dormancy, and host immunity.^[10]^

Due to the lack of histopathology and molecular evidence, we cannot conclude that the ciliary body seed in our patient was similar to the original choroidal tumor. Nevertheless, the possibility of a new ciliary body melanoma developing in the same eye as the choroidal melanoma exactly at the site of sclerotomy performed for FNAB is low, supporting our belief that the tumor in the ciliary body was a seed from the original choroidal tumor.

In summary, we report a rare case of FNAB-related needle tract seeding into the ciliary body with anterior chamber invasion and extraocular extension detected 20 years after pars plana transvitreal FNAB of choroidal melanoma that was performed with a 25-gauge needle. Long-term monitoring of all eyes with treated uveal melanoma is recommended, particularly at the scleral site of needle biopsy.

##  Financial Support and Sponsorship

The study was supported the by Eye Tumor Research Foundation, Philadelphia, PA (CLS). The funders had no role in the design and conduct of the study, in the collection, analysis and interpretation of the data, and in the preparation, review, or approval of the manuscript.

##  Conflicts of Interest

There are no conflicts of interest.
